# Identification of temperature regulated factors of *Campylobacter jejuni* and their potential roles in virulence

**DOI:** 10.3934/microbiol.2017.4.885

**Published:** 2017-11-07

**Authors:** Yue Tang, Shaun Cawthraw, Mary C. Bagnall, Adriana J. Gielbert, Martin J. Woodward, Liljana Petrovska

**Affiliations:** 1 Animal and Plant Health Agency, Woodham Lane, New Haw, Addlestone, Surrey KT15 3NB, UK; 2 School of Life Sciences, University of Warwick, Coventry, CV4 7AL, UK; 3 Food and Nutritional Sciences, University of Reading, Whiteknights, Reading RG6 6AP, UK

**Keywords:** proteomics, Q-Tof, QQQ protein quantitation, *Campylobacter* virulence, heat induced genes, *Galleria* model

## Abstract

*Campylobacter jejuni* is the major cause of bacterial gastroenteritis in man, while it is generally regarded as a commensal of the avian gut. Consumption and handling of contaminated poultry meat products are major risk factors for human infection. The body temperature in man (37 °C) and chickens (42 °C) differ markedly, and differential gene regulation and protein expression at different temperatures may in part explain the behaviour in the two hosts. We performed proteomics analyses with *C. jejuni* cells grown at 37 °C and 42 °C. Time-of-flight mass spectrometry (Q-Tof) analysis was carried out after samples were digested with the Filter-Aided Sample Preparation (FASP) method and peptides were fractionated by strong anion exchanges. Differentially regulated proteins were identified by Mascot and Scaffold analyses. Triple quadrupole (QQQ) mass spectrometer analysis confirmed that a total of 33 proteins were differentially regulated between 37 °C and 42 °C. Several upregulated proteins were selected for their corresponding gene knock-out mutants to be tested for their virulence in the *Galleria mellonella* model. To correlate with other tissue/animal models, the GADH mutant was selected for its reduced ability to colonize chickens. At 37 °C, the mutants of outer membrane protein Omp50 and Chaperone GroEL significantly increased virulence; while at 42 °C, the mutants of YceI, Omp50, and GADH reduced virulence against *Galleria mellonella* compared with the wild type strains. The results of current and previous studies indicate that GADH is a virulent factor in *G. mellonella* and a colonization factor in chickens. The workflow of this study may prove a new way to identify stress related virulent factors. The implications of these findings are discussed for pathogenesis in the model and other hosts.

## 1. Introduction

*Campylobacter jejuni* is a small, curved, Gram-negative bacterium that is the major cause of bacterial gastroenteritis in much of the world, including the UK [1]. It has generally been regarded as a commensal of the chicken gut [2],[3],[4], although it is known to induce pro-inflammatory responses [5] and recent data suggests it may induce strong inflammatory responses and cause diarrhoea in some commercial broiler breeds [6]. Consumption and handling of contaminated poultry meat products are thought to be major risk factors for human infection [4],[7], reportedly responsible for up to 80% of human campylobacteriosis cases [8]. *Campylobacter jejuni* is classified as one of the thermophilic campylobacters due to its ability to grow at higher temperatures than non-thermophilic species. This characteristic is reflected in its association with avian species where body temperatures are elevated (typically 39–42 °C) compared to those in mammals [9]. *C. jejuni* can be regularly isolated from a large range of pet and livestock animals but seems particularly to thrive in the caeca of poultry, where it can rapidly reach high numbers (typically 10^7^–10^9^ cfu/g) from an infective dose as low as 50 cfu [10]. This is in marked contrast to what is seen in immunologically naive humans, where typically exposure is followed fairly rapidly by severe gastroenteritis. In most cases the disease is self-limiting with illness lasting 5–14 days, although a number of severe sequelae, such as Guillain-Barré Syndrome, are also associated with infection [11].

Changes in temperature are known to affect expression of bacterial heat shock proteins (HSP). Several HSPs have been identified in *C. jejuni*, including the Lon, GroESL, DnaJ, DnaK and ClpB proteins [12]–[16]. The DnaJ protein has also been identified as a virulence factor in *C. jejuni* as a *dnaJ* mutant was unable to colonize chickens [16]. The genome of *C. jejuni* lacks a σ32 homologue that regulates expression of HSPs in *E. coli* [17] but contains two homologues to known heat shock response regulators: HcrA and HspR.

Thus understanding the biology of *C. jejuni* in human and chicken hosts should potentially aid the development of targeted intervention strategies. There are many differences between birds and humans, but one obvious difference is the higher body temperature of birds. It is probable that this temperature difference will be associated with differential regulation of genes that may encode proteins required for survival at the higher temperature or pathogenesis in humans. Therefore, in this study, we aimed to identify temperature responsive proteins that could play a role in host adaptation and virulence by performing Q-Tof mass spectrometry on *C. jejuni* grown at 37 and 42 °C. In addition, several mutants were selected according to their higher protein expression at 42 °C as potential virulence factors in the *Galleria* (wax worm) model.

## 2. Materials and Method

### 2.1. Bacterial strains and growth methods

All strains were stored at −80 °C in 1% (w/v) proteose peptone water containing 10% (v/v) glycerol until required. Strains were routinely grown from frozen stocks on 10% (v/v) sheep blood agar plates containing Skirrow's *Campylobacter* Selective Supplement (Oxoid) [18] at 42 °C in a microaerobic atmosphere [5% (v/v) N_2_, 7.5% (v/v) CO_2_, 7.5% (v/v) O_2_]. A loopful of overnight culture was placed into a biphasic Mueller-Hinton agar/broth and incubated in a microaerobic incubator at either 37 °C or 42 °C as previously described [19]. To ensure cells were recovered at similar stages of growth at each temperature bacterial growth was monitored by spectroscopy (OD 600 nm), with cells recovered by centrifugation when OD 600 was 0.6 (approx 18 hours for growth at 42 °C and 24 hours for 37 °C). All bacterial strains and mutants used in this study are shown in Table 1.

**Table 1.** *Campylobacter jejuni* strains used for proteomics and virulence tests.

Strain	Relevant characteristics	Source/reference
11168-GS	Wild type	APHA [20]
Cj420	11168-GS Δ YceI	*Campylobacter* Resource Centre of LSHTM
Cj117C	11168-GS Δ Omp50	*Campylobacter* Resource Centre of LSHTM
Cj414	11168-GS Δ GADH	*Campylobacter* Resource Centre of LSHTM
Cj1221	11168-GS Δ GroEL	*Campylobacter* Resource Centre of LSHTM
11168-O	Wild type	APHA [21]
Cj0289C	11168-O Δ Peb3	APHA

### 2.2. Protein extraction

Bacterial cells were recovered from the broth phase by centrifugation (5,000 × g for 10 min) and washed twice in sterile PBS. Cells were resuspended and disrupted by sonication (3 × 10 s) and cell-free extracts were recovered by centrifugation at 14,000 g for 10 min at 4 °C. Protein contents were determined by Coomassie Plus™ Protein Assay Reagent (Thermo Scientific) with BSA as the standard according to the Manufacturer's instructions.

### 2.3. Proteolytic digestion with trypsin and peptide fractionation by strong anion exchanges (SAX)

Digestion of the extracted proteins was carried out as described previously [22] according to the instructions of the Fasp Protein Digestion Kit which is based on filtering out undesirable substances during the sample preparations (Protein discovery, US). In brief, 0.4 mg of total protein resuspended in 30 µl UPX™ Universal Protein Extraction was heated at 99 °C for 10 min. Urea Sample Solution (200 µl) was mixed with protein samples in the Spin Filters and the filters were spun at 14,000 g for 15 min. The flow-through was discarded. A Volume of 10 µl 10× Iodoacetamide Solution and 90 µl Urea Sample Solution were added to the filters and mixed by vortexing for 1 min. The filters were kept in the dark for 20 min at room temperature before centrifuged at 14,000 g for 10 min. The Spin Filters were centrifuged again for 15 min after adding 100 µl Urea Sample Solution and this step was repeated twice. The flow through was discarded. Ammonium Bicarbonate Solution (100 µl) was added to the filters and the filters were centrifuged at 14,000 g for 10 min; this step was also repeated twice. Trypsin (5 µg, Promega, UK) in 100 µl Ammonium Bicabonate Solution was added to the filter. The mixture was vortexed and incubated at 37 °C for 18 hours. The filter was transferred to a fresh tube before peptides were collected at 14,000 g for 10 min. The collection of peptides was repeated after adding 100 µl Ammonium Bicabonate Solution to the filter. Finally, 100 µl 0.5 M NaCl was added and peptides were collected again. The peptide concentration was measured by NanoDrop 1000 (Thermo Scientific, USA) at 280 nm. Peptides were fractionated by strong anion exchange columns and fractionated peptides desalted using C18 columns as described previously [22].

### 2.4. LC-MS/MS analysis, spectrum identification and spectrum counting

The desalted peptide samples were analysed using a 1200 Series nano LC connected via a chipcube interface to a 6520 Q-ToF mass spectrometer (Agilent Technologies, UK). In brief, peptide samples (1 µg in 0.1% formic acid) were injected for LC-MS/MS analysis with a 120-min gradient from 3% (vol/vol) acetonitrile-0.1% (vol/vol) formic acid to 40% (vol/vol) acetonitrile-0.1% formic acid and from 3% (vol/vol) acetonitrile-0.1% (vol/vol) formic acid to 75% (vol/vol) acetonitrile-0.1% formic acid by using a high-capacity (160-nl) 150-mm C18 chip (Agilent Technologies, UK). The mass spectrometer was operated with mass ranges of 250 to 2,000 m/z for MS and 50 to 2,000 m/z for MS/MS at a constant flow rate of 500 nl/min. Acquisition rates were 3 spectra/s for MS and 5.01 spectra/s for MS/MS, and the mass reference was 922.009798 m/z. Mass accuracy was better than 2-ppm for MS and 5-ppm for MS/MS, and mass resolution was up to 20,000. Proteins were identified by Mascot (Version 2.3, Matrix, UK) from the NCBI non-redundant (nr) database (Release date: 28-03-2011) as described previously [23]. Identified proteins were quantified by spectral counting, using Scaffold (Version 2.6, Proteome Software, USA). The software was used to identify differentially expressed proteins using the *t* test with *P* value of less than 0.05 after normalization.

### 2.5. Peptide spectral library and analysis with Skyline

The Skyline software was used to find transitions for QQQ analysis and to quantify peptides/proteins after QQQ analysis. A spectral library was built using the Q-Tof data. For each differentially expressed protein, two peptides were selected for quantitation and each peptide was represented with three transitions, broken down products of the peptide. The transition list was exported to provide Multiple Reaction Monitoring (MRM), also called SRM (Selected Reaction Monitoring) information for QQQ mass spectrometry.

After QQQ runs the data were analysed with Skyline to identify retention times for each peptide. Once found, the information such as retention times was added to the transition list for QQQ mass spectrometry to run in dynamic MRM (dMRM) mode. The results of dMRM runs were analysed in Skyline to produce a quantity for each transition at a given retention time.

### 2.6. QQQ mass spectrometry quantification to validate

The peptide samples prepared with the FASP method were analysed using a 1200 Series nano LC connected to a 6410 QQQ mass spectrometer via a chipcube interface (Agilent Technologies, UK). Peptide samples (1 µg in 0.1% formic acid) were injected for LC-MS/MS analysis with a 10 minute gradient from 3% (v/v) acetonitrile, 0.1% (v/v) formic acid to 100% (v/v) acetonitrile, 0.1% formic acid using a Large Capacity Chip (160 nl) 150 mm C-18 (Agilent Technologies, UK).

### 2.7. Anova analysis with DanteR v1.01

DanteR (Pacific Northwest National Laboratory, US) is a R-based software package for omics data analysis [24]. The quantity of each transition was imported to DanteR from Skyline. The data were first quantile normalized. An Anova filter was applied to find statistically different transitions between 37 °C and 42 °C. The *P* value was set at 0.05.

### 2.8. Galleria survival tests

*G. mellonella* larvae were purchased from LiveFood UK and maintained on wood chips at 15 °C. The infection of larvae was carried out as previously described [19],[25]. Briefly, 10 µl of inoculum containing 1.0 × 10^6^ cfu of the selected strains grown at either 37 °C or 42 °C were injected into the top right pro-leg of each larva using a Hamilton® syringe. Larvae were incubated at 37 °C and survival determined 24 hours post-infection. Untreated and PBS injected controls were also included. Ten larvae were used in each experimental group and all experiments were carried out at least three times. Insects (including *Galleria mellonella*) are not covered by the Animals (Scientific Procedures) Act 1986 so no ethical approval is needed.

## 3. Results

### 3.1. Identification of proteins differentially expressed between 37 °C and 42 °C

Proteins extracted from *C. jejuni* 11168-GS bacterial cells grown either at 37 °C or 42 °C were trypsin digested and fractionated by strong anion exchange columns and subsequently analysed using a Q-Tof mass spectrometer. The protein lists were produced by Mascot. A total of 65 proteins were identified by Scaffold as differentially expressed when grown at 37 °C and 42 °C (Figure S1 and Table S1). Of these, 25 were upregulated and 35 were downregulated at 42 °C compared to expression at 37 °C and the maximal increase was 48.2 fold for PEB3 and the maximal decrease was 25.3 fold for cytochrome c1 subunit. At 42 °C, five chaperons, GroES, GroEL, Dnak, HSP90 and HtrA were up-regulated in expression (Table S1).

The differentially regulated proteins were validated using a QQQ mass spectrometer. MRM detection on a QQQ mass spectrometer provides sensitivity and selectivity for targeted peptides in a complex sample and also offers high precision in quantitation [26],[27]. Two peptides from each protein were selected for the analysis and each peptide was represented by three transitions. Six of the 12 proteins initially selected for the validation purpose using QQQ were found to be truly differentially expressed. They were 50S ribosomal protein L17, molecular chaperone DnaK, Gluconate 2-dehydrogenase subunit 3, major outer membrane protein, major antigenic peptide PEB3 and major antigenic peptide PEB4; whereas flagellin, heat shock protein 90, methyl-accepting chemotaxis protein, DNA-directed RNA polymerase beta' chain and F0F1 ATP synthase subunit beta were found false positives. This indicated that potentially half of the 65 identified proteins could be false positives. To eliminate all false positives, all 65 differentially expressed proteins were quantified by the QQQ. A total of 33 proteins were found to be truly differentially regulated (Table 2). Seventeen proteins including 4 chaperone proteins: GroEL, GroES, DnaK and HtrA were up-regulated at 42 °C; while 16 proteins down-regulated including some ribosomal proteins and elongation factors. The maximum increase was 2.91 fold for a putative periplasmic protein; whereas the maximal decrease was 3.69 fold for putative cytochrome C551 peroxidase (Table 2).

### 3.2. Virulence tests for mutants with the Galleria larvae

To test the hypothesis that thermal stress affects bacterial virulence, previously generated Ycel, Omp50, GADH, PEB3 and GroEL knock-out mutants in *C. jejuni* 11168 or 11168-O parental background strain were used to infect larvae of *G. mellonella*. To correlate results from other tissue/animal models, the GADH mutant was chosen as one of strains for its reduced ability to colonize chickens [28].

**Figure 1.**
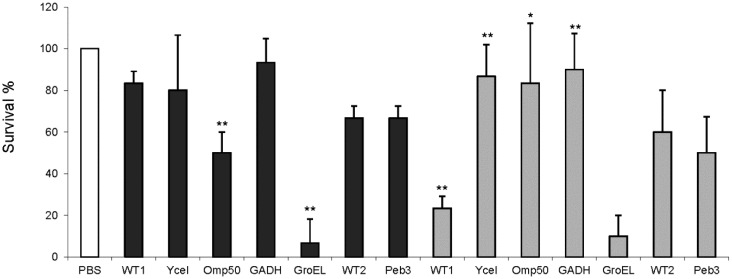
*G. mellonella* survival following challenge with *C. jejuni* wild type and knock-out mutants cultured at 37 °C (black) or 42 °C (grey) with *C. jejuni* doses of 10^6^ CFU/ml. Scored 24 hours post-infection. WT1, 11168-GS; WT2, 11168-O. The *t* test was carried out to compare the mutants with their parents. *, *P* < 0.05; **, *P* < 0.01.

Larval survival was recorded following challenge with WT1 (*C. jejuni* 11168-GS) and corresponding Ycel, Omp50, GADH and GroEL mutants or WT2 (*C. jejuni* 11168-O) and PEB3 mutant. The survival rate of *Galleria* larvae injected with WT1 of *C. jejuni* cultured at 37 °C was 83% after 24 hours, and survival rates of the larvae injected with the Ycel, Omp50, GADH and GroEL mutants were 80, 50, 93 and 6.7% respectively (Figure 1). The reduced survival of the larvae infected with the Omp50 and GroEL mutants was statistically significant: *P* = 0.0075 for Omp50 and *P* = 0.0005 for GroEL, suggesting that both mutants were more virulent in this model than the wild type parent strain (Figure 1).

The survival of larvae injected with WT1 cultured at 42 °C was significantly reduced compared to those injected with bacteria grown at 37 °C, 83.3% versus 23.3% (*P* = 0.0022), indicating the upregulation of virulence associated factors at 42 °C. The survival rates of larvae injected with Ycel, Omp50, GADH and GroEL mutants were 86.7, 83.3, 90 and 10%, respectively at 42 °C (Figure 1). Compared to the parental wild type strain, these rates were significantly increased for YceI (*P* = 0.0026), Omp50 (*P* = 0.024) and GADH (*P* = 0.0032), suggesting that they play a role in virulence against *G. mellonella* as without them the larvae survived better (Figure 1).

**Table 2.** *Campylobacter jejuni* proteins differentially regulated between 37 °C and 42 °C.

Protein ID	Protein name	Fold change at 42 °C	*P* value
gi|283954725	putative periplasmic protein	2.91	1.8E−02
gi|121612867	outer membrane protein Omp50	2.45	1.4E−02
gi|148926221	Chaperone GroEL	2.37	2.0E−02
gi|153952133	chaperone GroES	2.17	2.2E−02
gi|121612292	cytochrome c family protein	1.97	2.0E−02
gi|218561951	major antigenic peptide PEB3	1.96	4.8E−02
gi|86149030	Gluconate 2-dehydrogenase subunit 3	1.80	2.3E−02
gi|121613084	Chaperone DnaK	1.76	6.0E−03
gi|121613042	Chaperone HtrA	1.75	4.7E−02
gi|157414715	YceI	1.55	8.0E−03
gi|86150312	methyl-accepting chemotaxis protein	1.51	5.0E−03
gi|86149130	3-oxoacyl-(acyl-carrier-protein) reductase	1.51	4.0E−03
gi|86149619	thioredoxin	1.44	3.9E−02
gi|153952118	nonheme iron-containing ferritin ftn	1.42	4.1E−02
gi|118475164	uridylate kinase	1.38	4.3E−02
gi|121612116	pyridine nucleotide-disulphide oxidoreductase protein	1.34	4.2E−02
gi|118474190	Aconitate hydratase 2	1.28	2.1E−02
gi|153951800	50S ribosomal protein L17	−1.20	3.7E−02
gi|57237529	50S ribosomal protein L1	−1.20	4.1E−02
gi|148925859	DNA-directed RNA polymerase alpha chain	−1.25	4.3E−02
gi|218562794	30S ribosomal protein S2	−1.27	4.7E−02
gi|283955736	ribosomal protein L25	−1.33	2.0E−02
gi|148925693	Tungstate ABC transporter	−1.37	3.0E−02
gi|218563293	50S ribosomal protein L3	−1.38	3.1E−02
gi|153951395	cytochrome c553	−1.38	2.0E−02
gi|218562793	elongation factor Ts	−1.44	4.0E−02
gi|315638136	elongation factor P	−1.45	1.3E−03
gi|148926260	Ni/Fe-hydrogenase large subunit	−1.48	2.7E−02
gi|283955870	putative MCP-type signal transduction protein	−1.60	4.9E−02
gi|205356748	periplasmic nitrate reductase	−1.70	3.0E−02
gi|15741879	major antigenic peptide PEB4	−1.80	9.0E−03
gi|218562936	putative methyltransferase	−2.17	1.0E−03
gi|157414655	putative cytochrome C551 peroxidase	−3.69	8.0E−03

Note: APHA, Animal and Plant Health Agency; LSHTM, London School of Hygiene & Tropical Medicine.

## 4. Conclusion

In this study we applied quantitative proteomics to identify differentially expressed proteins in *C. jejuni* 11168 grown at 37 °C and 42 °C and to assess their potential role in host adaptation and fitness using *G. mellonella* as a surrogate *in vivo* model. The temperatures of 42 °C and 37 °C were chosen as representative of those in the intestines of chicken and humans respectively. The filter of Scaffold programme was set to identify as true positive samples with the *P* value less than 0.05. It is unclear from which steps the errors were generated, as when the two protein lists compiled using Q-Tof and QQQ were compared (Table 2 and Table S1), no patterns emerged. Some large fold change proteins, such as ubiquinol-cytochrome c reductase (∼25 fold), were subsequently found to be false positives; whereas some small fold change proteins, such as thioredoxin (1.2 fold) true positives by QQQ. The same is true for the *P* values. 50S ribosomal protein L9 with a small *P* value (*P* = 7.7E−04) was subsequently found a false positive; while YceI with a large *P* value (*P* = 0.049) a true positive. The false positives could come from Scaffold analysis or from Q-Tof or even Mascot as each step might produce some errors.

Similarly, the protein quantities were different in the protein lists produced by Q-Tof/Scaffold or QQQ/Skyline analyses. The Q-Tof/Mascot/Scaffold system identified differentially expressed between 1.2 and 48.2 fold for some of the proteins such as thioredoxin and Peb3 respectively; whereas the QQQ/Skyline system between 1.2 and 3.7 fold for 50S ribosomal protein L17 and putative cytochrome C551 peroxidase respectively. It is thought that a 3.7 fold change is more realistic than a 48.2 fold change for proteins. From this study we concluded that a validation step is crucial to test the reliability of data sets of protein profiling.

Several chaperone proteins, Dnak, GroES, GroEL and HtrA (Table 2) were found differentially upregulated at 42 °C consistent with previous studies [29]–[32]. The list of differentially regulated proteins identified in this study bears little resemblance to that reported in another proteomics study [33]. In this study, we used Q-Tof to get an initial list of differentially regulated proteins and then validated all of them with QQQ. Therefore, the methodology may be superior to a 2D gel only method as although the aims were similar, the methodologies used in the two studies are very different. One of the key issues of proteomic studies is solubilisation of samples. In our previous studies we found the Fasp method could identify many membrane proteins [22]. Our findings were in agreement with a gene expression study of *C. jejuni* [29] and other studies, such as the expression of heat shock genes in *Clostridium acetobutylicum* [30] and heat shock responses in *E. coli* and *Lactobacillus plantarum* [31],[32]. Five ribosomal proteins together with elongation factors Ts and P were downregulated at 42 °C (Table 2). The reduction of ribosomal proteins was also observed in a *C. jejuni* gene expression study where the author reasoned that a brief growth arrest allowed the cell to reshuffle energy devoted to an increased expression of genes involved in protective response and adaptation from 37 °C to 42 °C within a short interval [29]. However, in this study, cells were grown overnight at 42 °C, an optimal growth temperature [34]. Therefore, other explanations should be sought. For example, at 42 °C the cell may be more efficient so that there is no need to produce as many ribosomal proteins.

Infection models for *C. jejuni* include the ferret diarrhoeal model, colostrum-deprived piglet model and chick colonisation model [35],[36]. However, issues such as reproducibility, cost, ease of use, breeding and specialised training limit the use of these models [37]. We used the *Galleria* larvae (waxworms) as a model to determine potential roles in virulence for selected proteins identified in this study. The *G. mellonela* infection model has been reported previously for the study of *Campylobacter* [38] and also for the study of virulence factors in a number of other bacterial spp, including *Salmonella* *enterica*, *Staphylococcus aureus, Serratia marcescens, Pseudomonas aeruginosa* and Cryptococcus neoformans [39]–[43]. Furthermore, the model is also used to look at innate immune responses to pathogens since this branch of immunity is evolutionarily conserved amongst eukaryotes (unlike adaptive responses) so the innate antimicrobial responses in *Galleria* have similarities to those in higher organisms including man [44],[45]. Different degrees of attenuation were observed using the *Galleria* model when several knock-out mutants of *C. jejuni* 11168H in genes encoding potential virulence factors, such as LOS and cytolethal distending toxin [25]. The *C. jejuni* transcriptional regulator Cj1556 was found important for bacterial survival in Caco-2 human intestinal epithelial cells and J774A. A mouse macrophages and a mutant showed reduced virulence in *G. mellonella* [46]. Recently, Humphrey and co-workers found that a poultry isolate 13126 (sequence type 21) showed the greatest levels of extra-intestinal spread to the liver and is significantly more invasive than other isolates in human intestinal epithelial cells and gave the highest mortality in the *Galleria* infection model [47].

For the virulence test, it was expected to show more virulence towards *Galleria* at 37 °C to explain that the bacterium is seemingly a commensal at 42 °C in poultry and can cause severe gastroenteritis in human at 37 °C. However, the results were such that the wild type bacterium (WT1) was more virulent at 42 °C and several heat induced proteins, such as YceI, Omp50 and GADH were implicated in the virulence induction as the deletion mutants of those corresponding genes were less virulence at 42 °C compared with WT1 (Figure 1). In this study, at 37 °C both Omp50 and GroEL mutants killed more *Galleria* than the wild type parent strain. Although the reason for this is unclear, one possibility is that these proteins act as signals of infection to the *Galleria*. Initiation of insect (innate) immune responses begins with binding of pattern recognition proteins/receptors to conserved domains (pathogen-associated molecular patterns-PAMPs) located on the pathogen [48]. It is known that some members of the HSP family, including hsp60 (the eukaryotic homolog of GroEL), can stimulate the innate immune system directly [49]. Furthermore, bacterial GroELs are known to be antigenic in vertebrates [48],[49],[50], including *C. jejuni* infection in humans [50]. Thus it may be that *C. jejuni* lacking these proteins are not recognised as readily as wild-type bacteria resulting in delayed or decreased innate antimicrobial responses by the *Galleria*, so more larvae are killed. Further studies are needed to elucidate the roles (if any) of these in insect immunity, and whether a loss of other bacterial features involved in innate immune activation, e.g. flagella [51], would also lead to increased virulence in the model.

Of the three proteins described as virulence factors in this study (Figure 1), Omp50 is a pore-forming protein and sequence alignment shows weak identity with the major outer-membrane protein (MOMP) [52]. Its gene expression in *C. jejuni* is temperature-dependent and omp50::lacZ fusions showed that growth temperature control occurred at the transcriptional level [53].

Gluconate dehydrogenase (GADH) converts D-gluconate to 2-keto-D-gluconate. However, *C. jejuni* lacks an Entner-Doudoroff pathway so it cannot catabolize gluconate. It uses gluconate as an electron donor through GADH [28]. In *C. jejuni*, GADH is heat-induced and required for full colonization of avian but not mammalian hosts [28]. In this study, we also identified GADH as a heat responsive protein required for virulence in *Galleria* larvae.

The expression of YceI was induced at 42 °C (Table 2) and the mutant grown at that temperature displayed reduced virulence against *Galleria* (Figure 1). These properties are an addition to a long list of functions for this relatively unknown protein. YceI is a 22 kDa putative periplasmic protein which in *E. coli* binds to lipid [54] and in *Thermus thermophilus* to polyisoprenoid [55]. The protein crystal structure obtained at 2.9 Å resolution of *C. jejuni* YceI shows that it forms a dimer and binds to sulphate ion [56]. In *Helicobacter pylori*, YceI is secreted and is involved in adaptation to an acidic environment [57]. Other stress related functions include the induction of YceI by high pH and osmotic stresses [54],[58]. Thus, Ycel may be considered a general stress responsive protein. Some researchers suggest that YceI resembles “danger” infochemicals whose increased production by a bacterial subpopulation, becoming more resistant to bactericidal antibiotics, communicates a higher level of resistance to more sensitive members of the population of the same or different species [59].

In conclusion, a number of heat responsive proteins were identified by a Q-Tof mass spectrometer and subsequently confirmed by a QQQ mass spectrometer. Several knock-out mutants of heat-induced genes were selected to test in a virulence model. From these, Omp50, GADH and YceI were identified as virulence factors at 42 °C in the *Galleria* infection system. More studies are needed to determine if the *Galleria* infection model for *C. jejuni* can replace other tissue/animal models for identifying colonisation factors and virulence factors of vertebrates. The workflow developed in this study may prove a new way to identify stress related virulent factors.
