# Supplementary material for: Identification of temperature regulated factors of Campylobacter jejuni and their potential roles in virulence
Source: AIMS Microbiol. 2017 Nov 7;3(4):885–98. doi: 10.3934/microbiol.2017.4.885 (PMC6604965; doi:10.3934/microbiol.2017.4.885)
Supplement: Supplementary file 1 [file microbiol-03-04-885-s001.pdf]

AIMS Microbiology

AIMS Microbiology, 3(4): 885-898.
DOI: 10.3934/microbiol.2017.4.885
Received: 14 June 2017
Accepted: 24 October 2017
Published: 07 November 2017

http://www.aimspress.com/journal/microbiology

Research article

Identification of temperature regulated factors of *Campylobacter jejuni* and their potential roles in virulence

Yue Tang [1,*], Shaun Cawthraw [1], Mary C. Bagnall [2], Adriana J. Gielbert [1], Martin J. Woodward [3], and Liljana Petrovska [1,*]

[1] Animal and Plant Health Agency, Woodham Lane, New Haw, Addlestone, Surrey KT15 3NB, UK
[2] School of Life Sciences, University of Warwick, Coventry, CV4 7AL, UK
[3] Food and Nutritional Sciences, University of Reading, Whiteknights, Reading RG6 6AP, UK

* **Correspondence:** Email: yue.tang@apha.gsi.gov.uk; Liljana.petrovska@apha.gsi.gov.uk;
Tel: +44-2084152272.

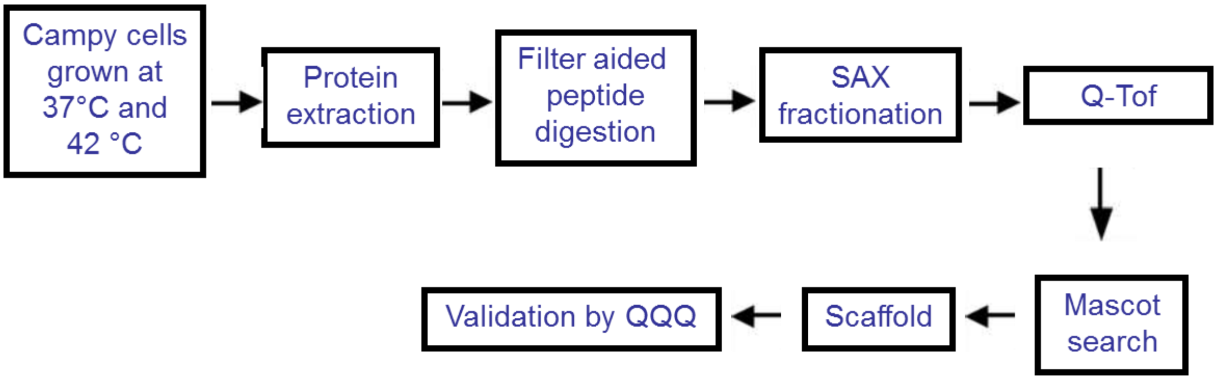

Figure S1. The workflow of mass spectrometry analyses.

Table S1. Differentially regulated proteins identified by Q Tof/Mascot/Scaffold.

ID	Protein	42/37 fold change	*P* value
gi|146386525	major antigenic peptide Peb3	48.2	7.8E−09
gi|118474190	Aconitate hydratase 2	26.5	1.9E−02
gi|121612995	cysteine desulfurase, putative	24.9	3.2E−03
gi|283954725	putative periplasmic protein	22.9	1.7E−02
gi|148926387	nifU protein like protein	9.8	1.2E−03
gi|153951038	F0F1 ATP synthase subunit beta	9.8	1.2E−03
gi|57238000	chemotaxis protein CheY	9.7	1.4E−03
gi|148926640	50S ribosomal protein L9	9.5	7.7E−04
gi|118475164	uridylate kinase	6.3	3.6E−05
gi|86149607	NifU family protein	6.0	8.9E−03
gi|157414697	serine hydroxymethyltransferase	5.3	2.2E−02
gi|121612116	pyridine nucleotide-disulphide oxidoreductase family protein	5.1	2.5E−02
gi|121612292	cytochrome c family protein	5.1	4.9E−02
gi|121612178	CjaA protein	5.0	1.1E−02
gi|86149130	3-oxoacyl-(acyl-carrier-protein) reductase	4.8	4.5E−02
gi|86150312	methyl-accepting chemotaxis protein	4.4	6.0E−03
gi|121613238	methyl-accepting chemotaxis protein	3.6	4.0E−02
gi|121613084	molecular chaperone DnaK	2.8	8.2E−03
gi|121612114	heat shock protein 90	2.4	1.0E−02
gi|218562948	flagellin	2.2	2.4E−02
gi|121613079	citrate synthase	2.1	4.4E−02
gi|148926221	Chaperone GroEL	1.8	1.0E−02
gi|153952118	nonheme iron-containing ferritin	1.8	1.1E−02
gi|218562871	major outer membrane protein Omp50	1.7	1.9E−02
gi|157414715	YceI	1.7	4.8E−02
gi|153952133	co-chaperonin GroES	1.5	6.3E−03
gi|86149030	Gluconate 2-dehydrogenase subunit 3 GADH	1.4	6.7E−03
gi|121613042	Chaperone HtrA	1.3	4.4E−02
gi|86149619	thioredoxin	1.2	1.4E−02
gi|121613736	hypothetical protein CJJ81176_1016	−1.2	7.3E−03
gi|153951395	cytochrome c553	−1.3	2.0E−02
gi|157415752	bacterioferritin	−1.4	7.7E−03
gi|205355703	50S ribosomal protein L15	−1.5	1.3E−02
gi|283955870	putative MCP-type signal transduction protein	−1.5	3.3E−02
gi|205356748	periplasmic nitrate reductase	−1.6	4.6E−20
gi|153952622	DNA-binding response regulator	−1.6	2.1E−02
gi|315930586	hydroxyisourate hydrolase	−1.6	3.8E−02

gi|148926260	Ni/Fe-hydrogenase large subunit	−1.7	3.0E−02
gi|148925906	OORA subunit of 2-oxoglutarate:acceptor oxidoreductase	−1.8	4.9E−03
gi|218562794	30S ribosomal protein S2	−1.9	1.5E−02
gi|86152788	glutamine-binding protein	−1.9	2.2E−02
gi|283955426	aspartate ammonia-lyase	−2.1	1.5E−02
gi|283955736	ribosomal protein L25	−2.4	3.0E−02
gi|121612137	2-oxoglutarate-acceptor oxidoreductase subunit OorB	−2.4	1.6E−03
gi|148925739	DNA-directed RNA polymerase alpha chain	−2.4	1.1E−02
gi|218563293	50S ribosomal protein L3	−3.0	2.5E−02
gi|118474959	2-oxoglutarate-acceptor oxidoreductase subunit OorA	−3.0	3.0E−02
gi|283956979	DNA-directed RNA polymerase alpha chain	−3.2	2.2E−02
gi|157414878	major antigenic peptide PEB4	−3.3	5.4E−06
gi|315637846	30S ribosomal protein S9	−3.7	2.3E−02
gi|153952132	50S ribosomal protein L18	−3.9	1.1E−02
gi|57237529	50S ribosomal protein L1	−4.5	1.9E−02
gi|148926940	putative periplasmic protein	−5.0	3.9E−02
gi|218562793	elongation factor Ts	−5.2	3.6E−02
gi|315929319	Putative uncharacterized protein	−6.3	1.1E−02
gi|148925859	DNA-directed RNA polymerase beta' chain	−6.7	8.3E−03
gi|218562936	putative methyltransferase	−7.6	1.6E−02
gi|148926980	rod shape-determining protein	−10.7	1.9E−03
gi|153951531	2-oxoglutarate-acceptor oxidoreductase subunit OorC	−10.7	1.9E−03
gi|153951800	50S ribosomal protein L17	−11.1	3.8E−03
gi|153951368	50S ribosomal protein L6	−12.7	8.7E−03
gi|148925693	Tungstate ABC transporter	−17.3	3.6E−04
gi|315638136	elongation factor P	−18.5	3.9E−02
gi|157414655	putative cytochrome C551 peroxidase	−25.3	1.7E−04
gi|121613025	ubiquinol-cytochrome c reductase, cytochrome c1 subunit	−26.0	2.9E−03

AIMS Press

© 2017 Yue Tang, et al., licensee AIMS Press. This is an open access article distributed under the terms of the Creative Commons Attribution License (http://creativecommons.org/licenses/by/4.0)
